# Association between Outdoor Air Pollution and Fatal Acute Myocardial Infarction in Lithuania between 2006 and 2015: A Time Series Design

**DOI:** 10.3390/ijerph20054549

**Published:** 2023-03-03

**Authors:** Vidmantas Vaičiulis, Jonė Venclovienė, Auksė Miškinytė, Rūta Ustinavičienė, Audrius Dėdelė, Gintarė Kalinienė, Dalia Lukšienė, Abdonas Tamošiūnas, Laura Seiduanova, Ričardas Radišauskas

**Affiliations:** 1 Health Research Institute, Lithuanian University of Health Sciences, Tilzes St. 18, 47181 Kaunas, Lithuania; 2 Department of Environmental and Occupational Medicine, Lithuanian University of Health Sciences, Tilzes St. 18, 47181 Kaunas, Lithuania; 3 Department of Environmental Sciences, Vytautas Magnus University, Donelaičio St. 58, 44248 Kaunas, Lithuania; 4 Institute of Cardiology, Lithuanian University of Health Sciences, Sukileliu Ave. 15, 50162 Kaunas, Lithuania; 5 Department of Health Politics and Management, School of Public Health, Asfendiyarov Kazakh National Medical University, Almaty 050000, Kazakhstan

**Keywords:** outdoor air pollution, fatal AMI, PM_10_, NO_2_, DOY, WHO MONICA

## Abstract

Background. Air pollution has a significant effect on human health and there is a broad body of evidence showing that exposure to air pollution is associated with an increased risk of adverse health effects. The main objective of this study was to assess the association of traffic-related air pollutants with fatal AMI during the ten-year period. Methods. The study was conducted in Kaunas city, where the WHO MONICA register included a total of 2273 adult cases of fatal AMI cases during the 10-year study period. We focused on the period between 2006 and 2015. The associations between exposure to traffic-related air pollution and the risk of fatal AMI were evaluated by using a multivariate Poisson regression model, RR presented per an increase in IQR. Results. It was found that the risk of fatal AMI was significantly higher in all subjects (RR 1.06; 95% CI 1.00–1.12) and women (RR 1.12; 95% CI 1.02–1.22) when the concentration of PM_10_ in the ambient air was increased 5–11 days before the onset of AMI, adjusting for NO_2_ concentration. The effect was stronger during spring in all subjects (RR 1.12; 95% CI 1.03–1.22), in men (RR 1.13; 95% CI 1.01–1.26), in younger-aged (RR 1.15; 95% CI 1.03–1.28), and in winter in women (RR 1.24; 95% CI 1.03–1.50). Conclusions. Our findings show that ambient air pollution increases the risk of fatal AMI, and this pertains to PM_10_ specifically.

## 1. Introduction

Air pollution has a significant effect on human health and there is a broad body of evidence showing that exposure to air pollution is associated with an increased risk of adverse health effects [1,2]. Ambient air pollution is one of the leading causes of mortality and morbidity worldwide [3]. A study of the Global Burden of Disease (GBD) estimated that total air pollution was responsible for approximately 6.7 million deaths in 2019, which included 2.3 million deaths due to household air pollution and nearly 4.2 million deaths attributable to fine particulate matter (PM_2.5_) pollution [4].

Cardiovascular mortality and morbidity account for two-thirds of the health effects of air pollution globally [2]. There is evidence that short-term exposure to air pollution is associated both with increased mortality from all-cause, respiratory, and cardiovascular diseases (CVDs), and with hospital admissions or emergency department visits [5,6]. A recent systematic review and meta-analysis on short-term exposure to air pollution and all-cause and cause-specific mortality showed that short-term exposure to PM_10_ (particles with an aerodynamic diameter less than 10 μm), PM_2.5_ (particles with an aerodynamic diameter less than 2.5 μm) and NO_2_ (nitrogen dioxide) was positively associated with all-cause and cardiovascular mortality [7].

Especially in recent years, a growing number of studies have reported that PM_10_ is associated with morbidity and mortality of circulatory system diseases. For example, a national study in the United States found that the multivariable-adjusted odds for the multiplicity of CVD outcomes increased by 1.15 times per 10 μg/m^3^ increase in PM_10_ [8]. In Rome, Italy, Alessandrini et al. reported that per inter-quartile range (IQR (19.8 μg/m^3^)) increase in PM_10_ concentration was associated with 2.6% higher hospitalization rates for cerebrovascular diseases [9]. In some Asian countries, such as Korea [10], Thailand [11], and Iran [12], similar results have also been reported.

The associations between short-term exposure to air pollution and CVDs have been investigated in multiple studies [13,14,15,16]. Many of these epidemiological studies were performed in various countries, such as the UK, Spain, Brazil, China, Iceland, Netherlands, Japan, and Austria, and have provided evidence that air pollution increases the risk of total CVD deaths [17,18,19,20,21,22,23,24,25,26,27].

However, there is a limited number of previous studies on the relationship between the short-term effect of air pollution and fatal AMI (Acute Myocardial Infarction), especially in the Baltic region and Northern Europe. Only a few studies in countries such as the USA, China, Hong Kong, South Korea, and Japan analysed the short-term effect of air pollution on fatal AMI [28,29,30,31,32,33].

There is relatively limited information on whether age or sex modifies the effect of air pollution on fatal AMI. Such effect modification has been seen in the associations between air pollutants (including PM_10_ and NO_2_) and CVD mortality, but the evidence on AMI remains inconclusive [34,35,36,37].

Furthermore, the results from the previous studies on air pollution and fatal AMI cannot be generalized to other populations or extrapolated to other regions, warranting more investigations in both local and global settings.

In addition, the research on the effects of air pollution on chronic diseases or their exacerbation must also consider meteorological conditions, in particular ambient air temperature, which may determine the concentration and composition of certain air pollutants and increase the risk of myocardial infarction [38]. For example, the solubility of air pollutants increases at lower air temperatures. According to previous studies, the incidence of AMI varies by season with the highest occurrence in winter [39]. There is previous epidemiological evidence of an association between cold outdoor temperatures and AMI [40,41].

Moreover, there is some evidence that cold climates can impact the characteristics of vehicle emissions. In cold weather, vehicles idle more, have high levels of cold-start emissions, including greenhouse gases, and have less effective exhaust filtration systems that can produce up to ten-fold more harmful vehicular emissions. In cities with cold climates, most urban short trips (e.g., 10–15 min) conclude before the vehicle has reached a fully warmed-up condition. For example, on a cold winter day, most vehicles are still in the cold phase after traveling a short distance, and exhaust after-treatment systems are operating at low-efficiency. There is still little evidence addressing those links in a cold climate [42,43].

Furthermore, the levels of air pollution in cold weather increase due to house heating combustion processes and meteorological conditions, such as thermal inversions, which trap and increase the levels of pollutants [44].

The study in China showed that the level of PM has a specific modification on the extreme cold effect [45]. There is evidence on the association between low temperatures and an increase in blood pressure, which is related to the stimulation of cold receptors in the skin, the increase in catecholamine levels of the sympathetic nervous system, and the constriction of blood vessels near the skin to reduce heat loss. Consequently, increased blood pressure can cause oxygen deprivation, myocardial ischemia, or arrhythmia, and become a risk factor for vascular spasms and atherosclerotic plaque ruptures leading to thrombosis. Due to these changes, people become more susceptible to the negative effects of particulate matter on CVD [46].

The general objective of this study was to assess the association of traffic-related air pollutants with fatal AMI during the ten-year period from 2006 to 2015. The associations between traffic-related air pollutants and the risk of fatal AMI were analysed during the all-study period and during different seasons. The specific objectives were to identify susceptible groups i.e., to assess effect modification by age, sex, and season.

## 2. Materials and Methods

The study was conducted in Kaunas city, which has a population of about 286,000 and is located in central Lithuania. In the present study, we focused on the ten-year period from 2006 to 2015. This study was carried out based on the data of the ischemic heart disease (IHD) register in Kaunas city, which was gathered in compliance with the recommendations of the World Health Organization (WHO) MONICA Project (Monitoring of trends and determinants in cardiovascular disease) [47]. The IHD register is run by a group of scientists at the Laboratory of Population Studies, Institute of Cardiology, the Lithuanian University of Health Sciences. The IHD register included Kaunas inhabitants aged 25–64 years, whose data were verified based on the WHO MONICA project protocol recommendations, and those aged ≥ 65 years, whose data were not verified. The main data sources were as follows: hospital statistical forms of discharged patients, hospital records, outpatient records, medical death certificates, and protocols of pathoanatomical and forensic investigation [47]. The study population comprised all Kaunas population aged 25 years and more who had fatal AMI (including patients who did not survive up to 28 days with a diagnosis of AMI) (ICD-10 I21-I22). While performing data analysis, risk factors of fatal AMI were evaluated in subject groups by sex and age. The study population was stratified into two age categories: (1) 25–64 years and (2) ≥65 years.

We used the mean daily concentrations of PM_10_ and NO_2_ from an air quality monitoring station. The data of concentrations were obtained from the air quality monitoring station of the Lithuanian Environment Protection Agency under the Ministry of Environment. The air quality monitoring station was located in Petrašiūnai, which reflects ambient air pollution of Kaunas city. It was assumed that the data from this station represented ambient exposure of the population. The concentrations of the pollutants were measured automatically on an hourly basis with continuous analysers based in the air quality monitoring station. Daily meteorological factors in the city of Kaunas were received from the Lithuanian Hydrometeorological Service under the Ministry of Environment. Kaunas meteorological monitoring station is located in the western part of the city (DMS coordinates 54°53′02.7″ N 23°50′09.2″ E). This monitoring station corresponds to quality control LST EN ISO 9001:2015 (Certificate No. 9000–493).

## 3. Statistical Analysis

Statistical data analysis was performed using IBM SPSS Statistics (Version 27). We conducted a time-series study to investigate the relationship between outdoor air pollution and daily mortality. As the daily numbers of fatal AMI *Y_t_* are the count variable, we assume that *Y_t_* followed a Poisson distribution with mean *λ_t_*, depending on predictor variables:

$$\ln\left(\lambda_{t}\right)=\beta_{0}+\beta_{1}X_{t}^{\left(1\right)}+\beta_{2}X_{t}^{\left(2\right)}+\ldots +\beta_{k}X_{t}^{\left(k\right)},$$

where *X*^(1)^, *X*^(2)^, …, *X*^(*k*)^ are covariates and *β*_1_, *β*_2_, …, *β_k_* are regression coefficients. In Poisson regression, the exp (*β_i_*) is defined as adjusted (for the remaining predictors) rate ratio (RR), *i* = 1, 2, …, *k*.

The associations between the PM_10_ and NO_2_ exposure and the risk of fatal AMI were evaluated by using a Poisson regression model [48,49]. In this model, we included the quadratic trend of the long-term time, the day of the week (categorical predictors with the categories Monday, …, Sunday, and Holidays, not coinciding with weekends), and the month as a categorical variable. Apart from this, we included the weather variable, affecting the risk of fatal AMI: air temperature two days before, both low and high atmospheric pressure (<1007 hPa and >1021 hPa), and high relative humidity (>88%) on the previous day [48]. The cut-offs of categorical variables were detected by using the classification and regression tree (CRT) method (Breiman et al. 1984). We found the effect of some large-scale patterns of atmospheric pressure anomalies and stratospheric winds having an impact on the climate in Lithuania on the risk of AMI [48] and stroke [49]. As the fatal AMI rate was lower during the west quasi-biennial oscillation (QBO) phase, then the presence west QBO was included in the model. We examined effects of daily PM_10_ and NO_2_ with single-day (with a lag of 0, 1, 2, …, 12) and multi-day lags. The multi-day lag was defined by using the RRs of the single-day lags. We assessed the effect of pollutants during the all-study period, during different seasons, and during different exposure levels. The level of exposure was assessed by using a mean 10-day value.

To assess the impact of environmental variables, we used adjusted rate ratios (RRs) per increase in the interquartile range (IQR) for air pollutants, their 95% confidence intervals (CI), and *p*-values in the multivariate Poisson regression model.

The analysis was performed for all patients and separately for men and women, and also for aged 25–64 and ≥65 years.

## 4. Results

The WHO MONICA register in Kaunas included a total of 2273 adult cases of fatal AMI cases during the ten-year study period. A total of 1507 (66.3%) of these occurred among men, while 766 (33.7%) cases were experienced among women. Table 1 shows the characteristics of the study population.

Over the study period, the mean daily temperature was 7.8 °C, and the mean daily air temperature ranged from −23.7 to 27.1 °C. The mean NO_2_ and PM_10_ concentrations in the residential locations were 16.8 and 30.7 (μg/m^3^), respectively. Table 2 shows the descriptive statistics of daily environmental variables in Kaunas during the study period.

**Figure 1:**
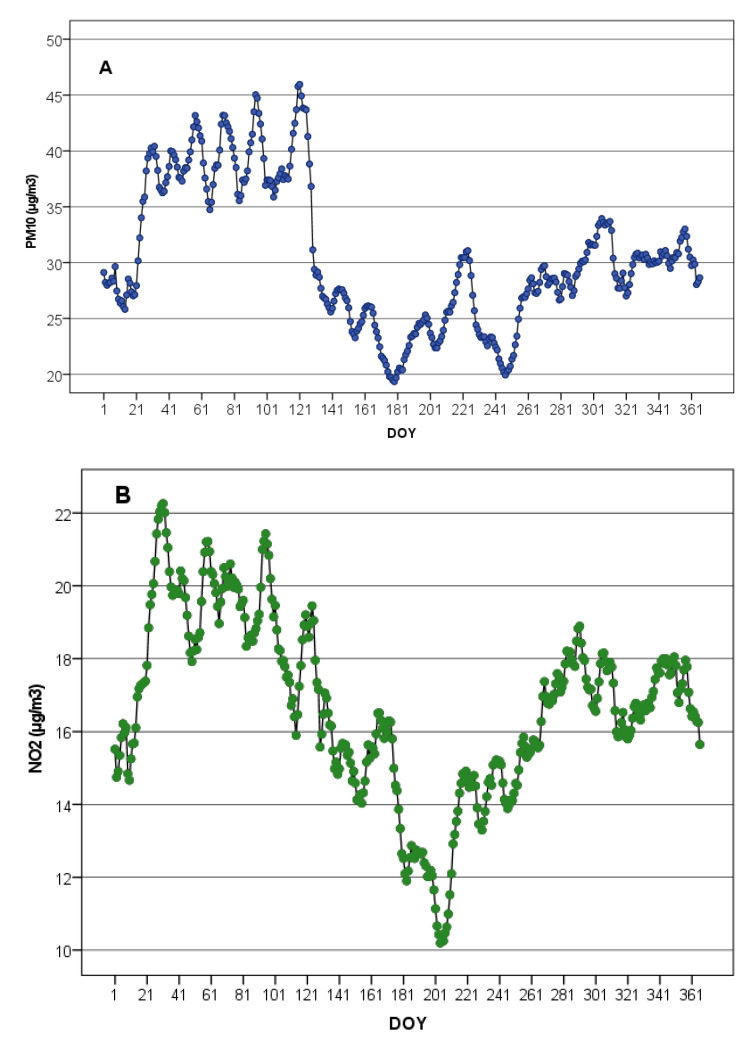
Seasonal dynamic of the 10-day average concentration of PM_10_ (**A**) and NO_2_ (**B**).

The highest mean 10-day values of PM_10_ were during the period of 23–133 day of year (DOY) (23 January–12 May) and the lowest values were during the period of 134–260 DOY (13 May–16 September) (Figure 1A). The seasonal dynamic of the 10-day average concentration of NO_2_ was similar to that of PM_10_ (Figure 1B).

We did not find any positive associations between the daily concentration of NO_2_ and the risk of fatal AMI. The associations between the daily concentration of PM_10_ and the daily fatal AMI with different lags are presented in Table 3. For single-day lags, RRs were >1 for lags 4–11. We found some statistically significant associations only at *p* = 0.9 confidence level (with a lag of 5–11 days). However, the effect of PM_10_ with a lag of 5–11 days was statistically significant during Spring and during the periods of a higher PM_10_ level (23–133 DOY). The associations between NO_2_ and the risk of death from AMI was non-significant during the periods of the different 10-day average concentration of NO_2_.

In the subgroup analyses conducted on specific age–sex-strata, the estimates between fatal AMI cases were similar (Table 4, Table 5 and Table 6). The results showed that women exposed to PM_10_ during 5–11 days before falling ill had a significantly higher risk of fatal AMI (RR = 1.09; 95% CI 1.00–1.19) (Table 4). After adjusting for NO_2_, the risk of fatal AMI in total age–sex-groups was statistically significant (RR = 1.06; 95% CI 1.00–1.12) (Table 4). Other results remained similar. In the subgroups, the effect of NO_2_ was non-significant.

According to the analysis during different seasons, the effect of PM_10_ was statistically significant during spring in the total group, men, and younger than 65 years, and during winter in women, especially 65 years and older (Table 5). As shown in Table 3, the effect of PM_10_ was statistically significant during the period of 23 January–12 May. During this period, the highest risk of fatal AMI was in the total group, among women, and ≤64 years old subgroups, (RR = 1.11; 95% CI 1.03–1.20), (RR = 1.14; 95% CI 1.01–1.29) and (RR = 1.13; 95% CI 1.02–1.24), respectively (Table 5).

In the analysis, during the period of 23 January–12 May, the mean concentration of PM_10_ 5–11 days before was categorized into the terciles. The cut-off of the terciles were 31.40 and 42.14 μg/m^3^. The first tercile was used as the reference. According to the results shown in Table 6, the risk in both terciles was similar, except for women. The highest risk of fatal AMI was among the total and women subgroups in the third tercile, (RR = 1.29; 95% CI 1.05–1.58) and (RR = 1.60; 95% CI 1.12–2.27), respectively.

## 5. Discussion

This population-based study in Kaunas showed positive associations between short-term exposure to PM_10_ and fatal AMI. The risk of fatal AMI was positively associated with the mean PM_10_ concentration on 5–11 days before the event in the two pollutants model (RR increase by 6%, *p* = 0.041); a stronger effect was observed in women (12%, *p* = 0.02). A stronger effect of PM_10_ on fatal AMI was observed in spring and during the period of late winter and the first two months of spring, which is the period of the highest PM_10_ levels. During this period, an increase in PM_10_ concentration with a lag of 5–11 days by IQR was associated with an increase in the risk of fatal AMI by 11% for all patients, by 14% for women, by 12% for men ≤ 64 years, and by 13% for aged 64 years (adjusting for NO_2_). Likewise, the risk of fatal AMI was higher in the third tercile (29%) than in the second tercile (24%) as compared to the first quartile. For men, especially for men aged < 65 years, the effect of PM_10_ was stronger in spring (RRs increase by 13%), but for women, this effect was stronger in winter (24%).

### 5.1. Synthesis with Previous Knowledge

Several previous studies in countries such as the USA, China, Hong Kong, South Korea, and Japan analysed the associations between air pollution and fatal AMI and many of them have found positive associations [28,29,30,31,32,33]. An especially bad situation is in the biggest country in Central Asia, Kazakhstan. Since the collapse of the Soviet Union, and the subsequent demolition of central heating infrastructure, and the dramatic fall in the economic level of population, also in the suburbs, a large fraction of the population in the suburbs now uses cheap fossil fuel for heating. This may explain the very high concentrations of ambient PM_2.5_ and PM_10_ during the cold season. A large fraction of the population in Almaty and other Kazakhstan cities is employed in outdoor jobs, and are likely exposed to high levels of particulate matter (PM) during the cold season [50]. However, the findings of a few previous studies from Asian countries (Japan and South Korea) showed that air pollution did not increase the risk of fatal AMI [31,32]. The big-volume study in the United Kingdom also did not find any significant association between death from AMI with short-term exposures to PM_10_ [51].

In many cases, our findings are in agreement with previous research and provide additional insights into similar studies in Lithuania, where some previous evidence exists on associations between traffic air pollution and health [52,53]. However, the short-term effect of PM_10_ on the risk of AMI was not investigated in these studies.

Moreover, no studies have comprehensively analysed the impacts of air pollution on fatal AMI in the three Baltic states. Most previous studies have focused on general chronic diseases or mortality [54,55,56], but not on fatal AMI.

According to some studies, potential biological mechanisms of how air pollution influences AMI can be related to oxidative stress, inflammation, abnormal regulation of the cardiac autonomic system, vascular dysfunction, thrombosis, and atherosclerosis [57,58,59]. Other studies reported that inhaling PM was associated with the activation of platelets and coagulation enzymes and might decrease the total myocardial flow significantly and increase coronary vascular resistance in animal studies [60,61]. There are many biological mechanisms, but oxidative stress is one of the main ones in explaining cardiovascular disorders caused by exposure to pollutants. Various pollutants lead to the development of oxidative stress, which leads to morbidity and mortality at various stages of the disease [62].

Short-term exposure to PM_10_ was also associated with increased homocysteine levels in smokers, but not in non-smokers [63]. Based on these results, it would appear that air pollution may determine short-term hypercoagulability, which in turn contributes to the increase in atherothrombotic cardiovascular events observed in the presence of high ambient concentrations of pollutants. The influence of ambient air pollution on inflammation, oxidative stress, blood coagulation, and autonomic function was also investigated in 76 young healthy adults from Taiwan by Chuang et al. [64], who found that increases in PM_10_, PM_2.5_, sulfate, nitrate, and O_3_ were associated with increases in C-reactive protein, 8-hydroxy-2′-deoxyguanosine (an oxidative stress marker), fibrinogen, plasminogen activator inhibitor-1, and decreased heart rate variability [65].

We determined a statistically significant delayed impact PM_10_ (with a lag of 5–11 days). This may be explained at first as due to the instigation of systemic pro-inflammatory response, and also vascular dysfunction, enhanced thrombosis, or coagulation potential [66]. We found a more pronounced effect of PM_10_ during the winter–spring period. During this period, morbidity of cold diseases is more frequent, and fewer people consume of vitamins, resulting in a reduction in the immune system and possibly enhancing pro-inflammatory responses.

We did not find any statistically significant associations between the risk of fatal AMI and NO_2_. Publications about NO_2_’s impact on AMI are controversial. Some publications evaluating the impact of chronic exposure of NO_2_ on blood pressure are showing that increases in NO_2_ concentrations are related to an increase in systolic blood pressure [67,68]. These studies were conducted in East Asian countries, where air pollution is significantly higher compared to the European region. Single studies in the European region and a large multi-cohort meta-analysis (ESCAPE) of the European study of cohorts for air pollution effects do not show a relation of NO_2_ concentration with CVDs [69,70].

Thus, it can be assumed that the differences in publications could be caused by different pollutant concentrations and higher levels of other pollutants. Our results show a non-linear association between the risk of fatal AMI and PM_10_ exposure: the risk in the second and the third PM_10_ terciles were similar. This is in agreement with other studies [30].

There is lack of short-term exposure evidence that age modifies the effect of air pollution on fatal AMI. One study conducted in China has shown that the association between NO_2_ exposure and MI mortality was significantly stronger in older adults [28]. Meanwhile, another study (long-term effect) in Sweden showed a stronger association in the younger age group (<65 years) [70]. No significant results by age were found in analysing PM_10_ association with fatal AMI [28,71].

Our study shows similar results to those of the previous study in Sweden. However, we did not find evidence of effect modification by age. Differences in our results may have been influenced by the different populations studied and the underlying comorbidities, as well as differences in methodology. The two studies that used methodology most similar to ours, one conducted in China and one in Sweden, both reported that the associations between air pollution and fatal AMI were different among both age groups. Considering that most of the previous studies did not perform statistical testing on whether their observed differences could be explained by chance, it seems that the question of the effect modification by age remains open.

There are just a few studies on the effect of modification by sex on the association between air pollution and fatal AMI. Several previous studies conducted in South Korea and China showed that the association between air pollution and fatal AMI was stronger among men than among women [29,32]. Another study from China showed that the effect estimates did not differ between men and women [28].

In our study, women seemed more susceptible to the effects of cold weather than men, but the difference between groups was not statistically significant. One of the explanations for these different results may be due to gender differences in comorbidity profiles [72,73] and behavioural factors, as well as differences in risk perception.

### 5.2. Strengths and Limitations

Our study has several strengths. The methodology of the Kaunas Ischemic Heart Disease Registry was based on unified protocols prepared for the WHO MONICA project, and the procedures for selecting research cases did not change during the study period. Another advantage of the study was the use of a case-crossover design, which is well-suited for assessing the short-term effects of risk factors over time and space, while also assessing the potential effects of other factors over time and controlling for confounders. Another relatively important strength of the study was the formal quantification of heterogeneity in subgroup values, which has not been analysed in other studies evaluating modification of the association between air pollution and AMI.

The limitations of the study could be attributed to the retrospective type of the Kaunas Ischemic Heart Registry, during which cases were registered and verified after the case left the treatment facility, and medical documentation in some cases could be lost, and the cases would remain unrecorded. However, given the relatively extensive data collection protocol, this number was very small and could not affect the results obtained. Cases of AMI at an older age (65 years and older) were also not verified in the study but were only registered according to the final clinical diagnosis. This limitation is common to all studies using the WHO MONICA criteria and did not significantly influence the results obtained. The decreasing percentage of autopsies over the past decades may have also introduced certain inaccuracies in the verification of a case according to the research protocol as a fatal case of death from ischemic heart disease. The effect of other severe concomitant pathology on the risk of fatal AMI cannot be excluded. Other lifestyle factors, such as harmful alcohol consumption, high stress levels, or psychosocial factors of the work environment at the time of case identification may have also been important, as fatal events were not recorded according to the registry methodology. We did not consider the impact of PM_2.5_, ozone, or noise pollution on the risk of fatal AMI. In addition, future studies should investigate individual environmental exposure levels in relation to the risk of AMI.

## 6. Conclusions

It was found that the risk of fatal AMI was significantly higher in all subjects and women when the concentration of PM_10_ in the ambient air was increased 5–11 days before the onset of AMI, adjusting for air NO_2_ levels. The stronger effect of PM_10_ was observed in the winter–spring period. Additional comprehensive studies analysing the immediate effects of air pollution on the risk of fatal MI will be needed in the future. Reducing pollution levels should be a key strategy to reduce the health burden of air pollution.

## Figures and Tables

**Table 1:** Characteristics of the study population of all fatal AMI cases in Kaunas during the study years 2006–2015, by age, sex, and season.

Characteristic	Winter, n (%)	Spring, n (%)	Summer, n (%)	Autumn, n (%)	All, n (%)
AMI fatal cases, all	645 (100)	564 (100)	518 (100)	546 (100)	2273 (100)
Men	430 (66.7)	361 (64.0)	348 (67.2)	368 (67.4)	1507 (66.3)
Women	215 (33.3)	203 (36.0)	170 (32.8)	178 (32.6)	766 (33.7)
25–64 years, all	385 (100)	324 (100)	301 (100)	331 (100)	1341 (100)
Men 25–64 years	329 (85.5)	267 (82.4)	258 (85.7)	281 (84.9)	1135 (84.6)
Women 25–64 years	56 (14.5)	57 (17.6)	43 (14.3)	50 (15.1)	206 (15.4)
≥65 years, all	260 (100)	240 (100)	217 (100)	215 (100)	932 (100)
Men ≥ 65 years	101 (38.8)	94 (39.2)	90 (41.5)	87 (40.5)	372 (39.9)
Women ≥ 65 years	159 (61.2)	146 (60.8)	127 (58.5)	128 (59.5)	560 (60.1)

**Table 2:** Descriptive characteristics of the daily environmental variables in Kaunas during the study period 2006–2015, by season.

Season	Variable	Range	Mean (SD)	Percentiles		
				25	50	75
All period	NO_2_ (μg/m^3^)	2–70	16.8 (8.5)	11	16	21
	PM_10_ (μg/m^3^)	4–185	30.7 (27)	18	27	38
Winter	Air temperature (°C)	−23.7–10.2	−2.5 (6.0)	−5.9	−1.0	1.7
	Wind speed (knots)	0–15	5.5 (2.3)	4.0	5.7	7.0
	Barometric pressure (hPA)	976–1050	1016 (12.2)	1008	1016	1024
	Relative humidity (%)	48–100	88 (7.6)	85	90	93
	NO_2_ (μg/m^3^)	2–68	18.7 (9.6)	12	17	23.5
	PM_10_ (μg/m^3^)	5–185	33.8 (22.0)	20	30	42
Spring	Air temperature (°C)	−15.2–23.1	7.3 (6.5)	2.8	7.6	11.9
	Wind speed (knots)	0–12.0	4.9 (2.0)	4.0	5.0	6.0
	Barometric pressure (hPA)	977–1047	1015 (9.1)	1009	1015	1021
	Relative humidity (%)	36–99	73 (12.8)	64	74	83
	NO_2_ (μg/m^3^)	2.0–70.0	18.1 (9.8)	11	16	24
	PM_10_ (μg/m^3^)	4–155	36.1 (21.9)	21	31	45
Summer	Air temperature (°C)	8.6–27.1	17.8 (3.3)	15.6	17.7	19.9
	Wind speed (knots)	0–11	4.2 (1.7)	3.0	4.0	5.0
	Barometric pressure (hPA)	988–1030	1014 (6.0)	1010	1014	1018
	Relative humidity (%)	42–98	74 (9.8)	68	75	81
	NO_2_ (μg/m^3^)	2–45	13.8 (6.8)	9	13	18
	PM_10_ (μg/m^3^)	4–69	23.9 (11.0)	16	22	31
Autumn	Air temperature (°C)	−11.6–23.0	8.1 (5.2)	4.5	7.8	12.4
	Wind speed (knots)	0–13.0	4.8 (2.0)	3.0	5.0	6.0
	Barometric pressure (hPA)	981–1040	1017 (9.7)	1010	1017	1024
	Relative humidity (%)	50–100	85 (8.2)	80	86	91
	NO_2_ (μg/m^3^)	2–41.0	16.7 (6.6)	12	16	21
	PM_10_ (μg/m^3^)	4–119.0	28.8 (15.5)	17	26	37

SD standard deviation; PM_10_ Particulate matter with an aerodynamic diameter less than or equal to 10 μm.

**Table 3:** The associations between the daily concentration of PM_10_ and the daily fatal AMI with different lags (adjusted RR per IQR increase).

PM_10_	RR	95% CI	*p*-Value
Lag0	1.03	0.98–1.08	0.281
Lag1	0.99	0.95–1.04	0.714
Lag2	0.99	0.95–1.04	0.701
Lag3	1.00	0.96–1.05	0.986
Lag4	1.02	0.97–1.06	0.496
Lag5	1.03	0.98–1.07	0.269
Lag6	1.04	0.99–1.08	0.111
Lag7	1.01	0.97–1.06	0.571
Lag8	1.02	0.97–1.07	0.460
Lag9	1.04	1.00–1.09	0.053
Lag10	1.04	0.99–1.08	0.126
Lag11	1.01	0.96–1.06	0.718
Lag12	0.99	0.94–1.03	0.561
Lags 5–11	1.03	1.00–1.06	0.094
Lags 5–11 spring	1.10	1.02–1.18	0.012
Lags 5–11 Winter	1.03	0.93–1.14	0.621
Lags 5–11, 134–260 DOY	0.98	0.84–1.15	0.863
Lags 5–11, <23 or >259 DOY	0.99	0.89–1.10	0.933
Lags 5–11, 23–133 DOY	1.08	1.01–1.16	0.017

**Table 4:** The effect of mean values of PM_10_ during 5–11 days before on the risk of fatal AMI during 2006–2015.

Characteristic	Non-Adjusting for NO_2_		Adjusting for NO_2_	
	RR (95% CI)	*p*	RR (95% CI)	*p*
Total	1.04 (099–1.10)	0.094	1.06 (1.00–1.12)	0.041
Men	1.02 (0.96–1.09)	0.555	1.03 (0.96–1.11)	0.427
Women	1.09 (1.00–1.19)	0.047	1.12 (1.02–1.23)	0.020
Men ≥ 65 years	0.97 (0.85–1.11)	0.685	0.98 (0.85–1.14)	0.754
Women ≥ 65 years	1.07 (0.97–1.18)	0.189	1.11 (0.99–1.23)	0.074
Men ≤ 64 years	1.03 (0.96–1.11)	0.365	1.05 (0.97–1.14)	0.273
Women ≤ 64 years	1.14 (098–1.34)	0.098	1.15 (0.96–1.37)	0.130
≤64 years	1.05 (0.98–1.12)	0.138	1.06 (0.99–1.14)	0.110
≥65 years	1.03 (0.95–1.12)	0.415	1.06 (0.97–1.16)	0.211

**Table 5:** The effect of mean values of PM_10_ during 5–11 days before on the risk of fatal AMI during spring, winter, and the period of the highest level of exposure (23 January–12 May).

Characteristic	Spring		Winter		23 January–12 May	
	RR (95% CI)	*p*	RR (95% CI)	*p*	RR (95% CI)	*p*
Total	1.12 (1.03–1.22)	0.011	1.05 (0.94–1.17)	0.413	1.11 (1.03–1.20)	0.006
Men	1.13 (1.01–1.26)	0.030	0.96 (0.83–1.10)	0.547	1.09 (0.99–1.20)	0.088
Women	1.10 (0.96–1.27)	0.185	1.24 (1.03–1.50)	0.022	1.14 (1.01–1.29)	0.026
Men ≥ 65 years	1.10 (0.87–1.01)	0.433	0.91 (0.68–1.23)	0.542	1.00 (0.81–1.23)	0.986
Women ≥ 65 years	1.05 (0.89–1.25)	0.555	1.25 (1.01–1.55)	0.044	1.13 (0.98–1.31)	0.087
Men ≤ 64 years	1.13 (1.00–1.28)	0.049	0.97 (0.83–1.14)	0.731	1.12 (1.00–1.25)	0.049
Women ≤ 64 years	1.23 (0.96–1.58)	0.111	1.20 (0.82–1.76)	0.355	1.19 (0.95–1.50)	0.146
≤64 years	1.15 (1.05–1.28)	0.016	1.00 (0.86–1.16)	0.990	1.13 (1.02–1.24)	0.012
≥65 years	1.07 (0.93–1.23)	0.348	1.12 (0.94–1.33)	0.218	1.08 (0.96–1.22)	0.195

RR per increase IQR, additionally adjusted for NO_2_.

**Table 6:** RR with 95% CI in terciles of mean concentration of PM_10_ during 5–11 days before during the period of 23 January–12 May, additionally adjusting for NO_2_.

Characteristic	RR (95% CI)	*p*	RR (95% CI)	*p*
	* Second Tercile		* Third Tercile	
Total	1.24 (1.02–1.51)	0.026	1.29 (1.05–1.58)	0.013
Men	1.15 (0.91–1.46)	0.233	1.16 (0.90–1.49)	0.232
Women	1.46 (1.04–2.05)	0.031	1.60 (1.12–2.27)	0.009
≤64 years	1.21 (0.93–1.56)	0.154	1.29 (0.98–1.68)	0.063
≥65 years	1.30 (0.97–1.75)	0.081	1.30 (0.95–1.78)	0.109

* The first tercile as the reference.

## Data Availability

The health data was obtained from the Institute of Cardiology under the Lithuanian University of Health Sciences. The data is not accessible online. The weather and pollution data were obtained from the Lithuanian Hydrometeorological Service and the Environmental Protection Agency, respectively. The data is not accessible online. Health data requests can be emailed to: dalia.luksiene@lsmuni.lt.
